# Tobacco smoking, alcohol drinking and Hodgkin's lymphoma: a European multi-centre case–control study (EPILYMPH)

**DOI:** 10.1038/sj.bjc.6603229

**Published:** 2006-07-04

**Authors:** H Besson, P Brennan, N Becker, S De Sanjosé, A Nieters, R Font, M Maynadié, L Foretova, P L Cocco, A Staines, M Vornanen, P Boffetta

**Affiliations:** 1 International Agency for Research on Cancer, 150 cours Albert Thomas, 69372 Lyon Cedex 8, France; 2 Division of Clinical Epidemiology, German Cancer Research Centre, Heidelberg, Germany; 3 Department of Epidemiology and Cancer Registry, Catalan Oncology Institute, Barcelona, Spain; 4 Unit of Biological Haematology, Dijon University Hospital, Dijon, France; 5 Department of Cancer Epidemiology and Genetics, Masaryk Memorial Cancer Institute, Brno, Czech Republic; 6 Institute of Occupational Medicine, University of Cagliari, Cagliari, Italy; 7 Department of Public Health, University College Dublin, Dublin, Ireland; 8 Department of Pathology, Tampere University Hospital, Tampere, Finland

**Keywords:** Hodgkin's lymphoma, tobacco, alcohol, Europe

## Abstract

We analysed the effects of tobacco and alcohol in the aetiology of Hodgkin's lymphoma (HL), based on 340 cases and 2465 controls enrolled in Spain, France, Italy, Germany, Ireland and Czech Republic, between 1998 and 2004. Current smokers showed a significantly increased odds ratio (OR) of HL of 1.39 (95% confidence interval (CI)=1.04–1.87). Analyses were also conducted separately for subjects younger than 35 years (179 cases) and for older subjects (161 cases). For subjects below age 35, no association was observed between tobacco and HL, whereas for older subjects, ever-smokers experienced a doubled risk of HL as compared to never smokers and the OR of HL for current smoking was 2.35 (95% CI=1.52–3.61), with suggestion of a dose–response relationship. A protective effect of alcohol was observed in both age groups. The OR for ever-regular drinking was 0.58 (95% CI=0.38–0.89) for younger subjects and 0.50 (95% CI=0.34–0.74) for older subjects. There was no evidence of interaction between tobacco and alcohol. Our results are consistent with previous studies, suggesting a protective effect of alcohol on HL. An effect of tobacco was suggested for HL occurring in middle and late age, although this finding might have occurred by chance.

Hodgkin's lymphoma (HL) accounts for approximately 30% of all lymphomas (Jaffe *et al*, 2001). In developed countries, its incidence peaks around age 25–30 and then again after age 60 (Cartwright and Watkins, 2004). To date, the two main known risk factors of HL are infection with Epstein–Barr virus (EBV) with late age of infection being potentially important (International Agency for Research on Cancer (IARC), 1998) and infection with human immunodeficiency virus (IARC, 1996).

Sixteen epidemiological studies have investigated the effect of tobacco smoking on HL (Hammond and Horn, 1958; Newell *et al*, 1973; Paffenbarger Jr *et al*, 1977; Williams and Horm, 1977; Abramson *et al*, 1978; Matthews *et al*, 1984; Bernard *et al*, 1987; McLaughlin *et al*, 1995; Siemiatycki *et al*, 1995; Adami *et al*, 1998; Miligi *et al*, 1999; Stagnaro *et al*, 2001; Briggs *et al*, 2002; Gallus *et al*, 2004; Glaser *et al*, 2004; Nieters *et al*, 2005). Out of the 11 reporting an increased risk (Hammond and Horn, 1958; Paffenbarger Jr *et al*, 1977; Williams and Horm, 1977; Matthews *et al*, 1984; McLaughlin *et al*, 1995; Siemiatycki *et al*, 1995; Adami *et al*, 1998; Stagnaro *et al*, 2001; Briggs *et al*, 2002; Glaser *et al*, 2004; Nieters *et al*, 2005), three presented significant results (McLaughlin *et al*, 1995; Briggs *et al*, 2002; Nieters *et al*, 2005), among which one is part of our research (Nieters *et al*, 2005). These studies were characterised by small sample size, 10 of the 15 studies being based on less than 200 case–control pairs. As a consequence, HL is not considered as a smoking-related cancer (Jaffe *et al*, 2001; IARC, 2002). The literature concerning the potential link with alcohol drinking is based on four case–control studies (Williams and Horm, 1977; Bernard *et al*, 1987; Tavani *et al*, 1997; Nieters *et al*, 2005), all reporting an apparent protective effect of alcohol. To clarify further the potential association between tobacco, alcohol and HL, we have investigated these associations in a European multicentre case–control study (Epilymph) in which the serological EBV status was tested in a subsample of subjects.

## PATIENTS AND METHODS

### Subjects

Our investigation was conducted in six European countries: Czech Republic, France, Germany, Ireland, Italy and Spain. Recruitment of histologically or cytologically confirmed incident cases of lymphoma, among which 340 were HL, began in 1998 and ended in 2004. Cases were classified according to the World Health Organization classification (Jaffe *et al*, 2001), with a central review of 20% of the slides from each centre in order to confirm the reliability of diagnoses reported on confirmation of HL diagnoses.

During the same period, 2465 controls were recruited. In Italy and Germany, controls were drawn from a random sample of the population registers of the recruitment areas. These population-based controls were matched to cases on sex, age (5-year intervals) and residence area. In other countries, controls were hospitalised subjects suffering from infectious and parasitic (17.6%), mental and nervous (14.6%), circulatory (8.7%), digestive (7.1%), endocrine and metabolic (4.1%), respiratory (3.9%) and several other conditions (33.2%) excluding cancer. The remaining 10.8% of diagnoses were unspecified. These controls were matched to cases on age (5-year intervals), sex and region.

The matching was group-based in France, Ireland, Italy and Spain, whereas controls were individually matched to cases in Germany and Czech Republic. The participation rates for hospitalised and population-based controls were, respectively, 81.2 and 51.5%, whereas among cases it was 87.7%. All cases and controls were at least 17 years of age. The study was approved by the relevant ethical committees.

### Data collection

A standard questionnaire was translated in the language of each country and administered to all patients by locally trained interviewers. Questions covered sociodemographic variables, occupational, reproductive, family and medical history, ultraviolet radiation exposures as well as lifestyle factors, including alcohol drinking and tobacco smoking. In Germany, a slightly different questionnaire on alcohol drinking was used (Nieters *et al*, 2005). In addition, for 1033 controls and 196 cases of HL (coming from four countries: Spain, France, Germany and Italy), two observers independently and blind to the disease status tested the EBV status in serum using an enzyme-linked immunosorbent assay.

Extensive information about tobacco smoking was collected for all patients, including smoking status, type of tobacco (black or blond), age at start, duration (years) and number of cigarettes smoked per day for ever-smokers (ex- and current smokers combined). An ever-smoker was defined as someone smoking at least one cigarette per day for 6 months and a current smoker was defined as a participant who reported smoking 2 years before the date of interview. The number of cigarettes smoked per day was based on the average consumption. Quantitative variables were categorised according to tertiles of the distribution of smokers among young and old controls separately.

Description of alcohol consumption including type of beverage drunk (beer, wine or spirits), volume consumed per month, age at start and duration (years) of drinking was collected for regular drinkers defined as people declaring a daily consumption lasting over 6 months. In Germany, a regular drinker was defined as a man consuming more than 2 g of alcohol per day. The limit was 0.5 g day^−1^ for women. Information related to beer, wine and spirits was collected separately and then summed up in order to estimate consumption for all alcohol intake combined. Lifetime consumption (kilograms) was calculated by summing the product obtained for each period of consumption. (Lifetime alcohol consumption (kilograms) = ∑_i_12*d*_*i*_*c*_*i*_*w*_*i*_+∑_*j*_12*d*_*j*_*c*_*j*_*w*_*j*_+∑_k_12*d*_*k*_*c*_*k*_*w*_*k*_, where *d* is the duration (years), *c* the amount (l month^−1^), *w*_*i*_=0.004 kg l^−1^, *w*_*j*_=0.01 kg l^−1^, *w*_*k*_=0.032 kg l^−1^, *i*=beer, *j*=wine and *k*=spirit) (IARC, 1988). Monthly amount of alcohol was calculated in the same way divided by the duration of drinking (in months). Quantitative variables were categorised according to tertiles of the distribution of drinkers among young and old controls separately. In Germany, because of the use of a slightly different questionnaire, the following alcohol drinking variables were not available: age at start, duration and lifetime consumption.

### Statistical analysis

To allow for possible aetiological differences between HL in young *vs* older people (Jarrett, 2002), all analyses were stratified into two categories (subjects younger than 35 years and subjects 35 years or older). Odds ratios (ORs) of HL and their corresponding 95% confidence intervals (CIs) were computed for alcohol drinking and tobacco smoking by using logistic regression models. Odds ratio for smoking-related variables were adjusted for sex, age (5-year intervals), educational level (in three categories) and consumption of alcohol per month (in three categories). Similarly, OR for alcohol drinking were adjusted for sex, age (5-year intervals), educational level (in three categories) and smoking status (in three categories). Never smokers were the reference category for the analyses on tobacco smoking and never-regular drinkers were the reference category for each of the analyses on alcohol drinking. Linear trends were calculated by including categorical exposure variables in the multivariate regression models, whose significance was tested by the Wald test. In order to test the heterogeneity of risk by sex and by centre, an interaction term was added to the regression model and its significance was tested by the likelihood ratio statistic. Breslow and Day tests were performed to test homogeneity between EBV-positive HL *vs* EBV-negative HL as well as between the two main histological types of HL, nodular sclerosis (HLNS) and mixed cellularity (HLMC). Additionally, to test the interaction between smoking and alcohol, a binary interaction term was included. Analyses were conducted using Statistical Analysis System software version (SAS Institute Inc, 2004).

## RESULTS

The 340 identified HL were composed of 24 nodular lymphocyte predominant HL and 316 classical HL, which were made up of 193 HLNS, 69 HLMC, nine lymphocyte-rich HL, four lymphocyte-depleted HL and 41 classical HL not otherwise specified (nos.). As reported in Table 1, in both age groups, over 90% of the cases were diagnosed as classical HL. Cases of HLNS, which is the main histological subtype, represented two-thirds of the cases less than 35 years old and 45% of the older cases. With respect to sociodemographical variables, the younger cases were similar to the controls, whereas older subjects were more educated than controls.

In Table 2, detailed results of the analysis of the effects of smoking are presented regardless of age and by age for HL overall and for HLNS. Overall, tobacco smoking increased risks of HL with an OR of 1.33 (95% CI = 1.02−1.74) for ever-smokers and an OR of 1.39 (95% CI = 1.04−1.87) for current smokers. Among younger subjects, no association was observed between smoking and HL. Among older subjects, increased risks of HL overall were observed with an almost doubled OR for ever-smokers and an OR of 2.35 (95% CI = 1.52−3.61) for current smokers. A trend in OR was observed with cumulative smoking expressed as pack-years. On the other hand, no dose – response relationship was observed with cigarettes smoked per day, age at start or duration of smoking. There was no evidence of heterogeneity of the effect of tobacco on the development of HLMC *vs* HLNS (*P* = 0.87). For both histological subtypes, current smokers displayed a more than two-fold increased risk of HL but no dose – response relationship was observed.

Table 3 reports the results of HL risk for alcohol drinking. Overall, a protective effect was observed with an OR of 0.61 (95% CI = 0.43−0.87) for ever-regular drinkers, reaching 0.56 (95% CI = 0.42−0.74) when cases and controls from Germany were included (data not shown). Among younger subjects, a protective effect of ever-regular drinking on HL was observed. Inclusion of cases and controls from Germany resulted in an OR of 0.58 (95% CI = 0.38−0.89) for ever drinking. No trend was observed for increasing level, except for duration of drinking. The protective effect was observed for HLNS but not for HLMC (data not shown). Results on alcohol drinking among older subjects were similar to those observed among young subjects; in particular, the inclusion of cases and controls from Germany resulted in an OR of 0.50 (95% CI = 0.34−0.74) for ever drinking. A dose – response relationship was suggested with lifetime consumption of alcohol.

There was no evidence of interaction between ever smoking and ever drinking status among either young (*P*=0.72) or older subjects (*P*=0.40). Among the older subjects, the increased risk of HL owing to smoking was equal to 2.66 (95% CI = 1.06−6.65) for regular drinkers and 2.11 (95% CI = 0.98−4.57) for nonregular drinkers, and no evidence of heterogeneity of the effect of tobacco was observed on the development of EBV-negative HL *vs* EBV-positive HL (*P*=0.53). There was no evidence of heterogeneity looking at the effect of alcohol on development of EBV-negative HL *vs* EBV-positive HL for either young (*P*=0.54) or older subjects (*P*=0.25).

## DISCUSSION

This European study involving 340 cases of HL and 2465 controls suggested that among subjects older than 35 years but not among younger subjects, tobacco smoking increased the risk of HL. On the other hand, alcohol drinking was suggested to decrease the risk of HL in both young and old subjects. These results have little support from the epidemiological literature, although this is characterised by a limited number of studies investigating the effect of alcohol and stratifying the effect of tobacco by age. No interaction between smoking status and alcohol consumption on the risk of HL was present, which to our knowledge has never been investigated previously.

Our study has several methodological strengths. The questionnaire was administered in the same manner to all subjects, cases and controls. All HL patients are incident cases and their diagnoses have been histologically or cytologically confirmed. Diagnoses of 97 cases were reviewed centrally: of them, 83 (85.6%) cases were validated, 13 (13.4%) were reclassified between HL subtypes and one was inconclusive. The distribution of the different histological subtypes of HL was comparable to those reported worldwide (Jaffe *et al*, 2001). Hospital-based controls were recruited in the same hospitals or hospitals from the same recruitment areas as the cases; with regards population-based controls, in addition to matching for age and sex, they were frequency matched for residential area insuring that controls were drawn from the same source population as the case series. The two centres recruiting population-based controls reported similar results as the four centres recruiting hospital-based controls.

Nonetheless, consideration must be given to potential limitations of our investigation. Cases and controls were characterised by refusal rates of 12.3 and 31%, respectively. If nonparticipants drink or smoke differently than included subjects, this may lead to over- or underestimating the true ORs. As participants were asked to report habits for many years before interview, there may have also been some exposure misclassification. If this misclassification was undifferential, this would dilute observation of associations and dose – response relationships, whereas if this misclassification was differential, it would exaggerate or decrease observation of associations. Furthermore, alcohol and even more tobacco are well-known risk factors of cancer. This may result in higher recall among cases than controls, potentially leading to overestimating the effect of risk factors and to underestimating the protective effects. In addition, the EBV status at our disposal was a serological one, which cannot necessarily be interpreted as part of the malignant process.

The evidence of a positive association between tobacco smoking and HL risk among subjects over 35 years old was strengthened by our observation of a dose–risk relationship for number of pack-years, although there was no significant heterogeneity in risk by age of smoking duration using the same categories of smoking duration (*P*=0.11). The difference of risk of HL observed by age might not be a consequence of longer smoking duration among older subjects. For most tobacco-related cancers, a carcinogenic effect of tobacco is also apparent among young subjects (IARC, 2002). Our data add further evidence that the young-onset HL and the late-onset HL are two different diseases with likely different aetiological pathways.

Although an association between smoking and HL has been reported or suggested by several studies (Hammond and Horn, 1958; Paffenbarger Jr *et al*, 1977; Williams and Horm, 1977; Matthews *et al*, 1984; McLaughlin *et al*, 1995; Siemiatycki *et al*, 1995; Adami *et al*, 1998; Stagnaro *et al*, 2001; Briggs *et al*, 2002; Glaser *et al*, 2004), HL has not been regarded as a smoking-related cancer (Jaffe *et al*, 2001; IARC, 2002). Out of the five previous investigations stratifying the analyses by age or restricting inclusion to subjects over 35 years old, two American studies reported significant increased risks of HL owing to smoking (McLaughlin *et al*, 1995; Briggs *et al*, 2002). One was a cohort study composed of 248 046 US Veterans with 3 000 000 person-years of observation accumulated, which reported a significant relative risk of 1.5 for current smokers (McLaughlin *et al*, 1995). The other was a male population-based case–control study, in which current smokers over 42 years old had a significant doubled risk of HL (Briggs *et al*, 2002). However, no heterogeneity in risk was observed by comparing subjects younger *vs* older than 42 years (Briggs *et al*, 2002). The three other studies whose results were based on older subjects were a Canadian case–control study (Siemiatycki *et al*, 1995) and two cohort studies conducted in Sweden (Adami *et al*, 1998) and the USA (Hammond and Horn, 1958); these reported insignificant increased risk of HL owing to smoking. Four studies investigated the dose–response relationship, but none stratified analyses by age (Stagnaro *et al*, 2001; Briggs *et al*, 2002; Gallus *et al*, 2004; Glaser *et al*, 2004).

The inverse association between alcohol and HL is consistent with results from three case–control studies carried out in Italy (Tavani *et al*, 1997), United Kingdom (Bernard *et al*, 1987) and USA (Williams and Horm, 1977), which reported reduced OR of HL ranging from 0.5 to 0.9. Our results are weakened by lack of observation of a dose—response, except with lifetime consumption for which a dose–response relationship was suggested among both younger and older subjects. None of the previous studies investigated in detail the dose–risk relationship with alcohol drinking.

It is possible that alcohol is proapoptotic and acts like aspirin (Chang *et al*, 2004) by inhibiting the transcription nuclear factor-*κ*B (Guizzetti *et al*, 2003; Saeed *et al*, 2004). Nuclear factor-*κ*B initiates transcription of antiapoptotic genes such as *bcl-2* and has been found to be constitutively activated in the Hodgkin and Reed–Sternberg (HRS) cells (Kuppers and Hansmann, 2005) blocking in this manner the apoptotic pathway. In contrast, inhibition of active NF-*κ*B decreases proliferation and causes spontaneous apoptosis of HRS cells (Bargou *et al*, 1997; Hinz *et al*, 2001; Izban *et al*, 2001). Within the subsample with documented EBV, subjects with negative EBV status did not drink more than those positive for EBV status (data not shown). On the other hand, the inverse association between alcohol drinking and HL might also be owing to unmeasured confounding factors.

In conclusion, on the basis of the findings of this study, one of the largest on this subject in Europe, smoking increases the risk of HL among subjects older than 35 years but not among younger subjects. The results also suggest that regular alcohol drinking is associated with a decreased risk of HL in both age groups. However, our findings must be treated with caution and further evidence is needed, especially on alcohol drinking.

## Figures and Tables

**Table 1:** Sociodemographic characteristics of HL cases and their controls by age groups

	**Controls *n*=2465**		**Cases *n*=340**	
	***n***	**%**	***n***	**%**
**Subjects <35 years old**				
*Histology*				
HLNS			121	67.6
HLMC			29	16.2
Other classical HL			19	10.6
Unclassical HL			10	5.6

*Sex*				
Male	162	51.9	90	50.3
Female	150	48.1	89	49.7

*Age (years)*				
<25	106	34.0	66	36.9
≥25 and <30	99	31.7	71	39.7
≥30 and <35	107	34.3	42	23.5

*Educational level*				
Missing data	1		0	
Low	55	17.7	29	16.2
Medium	192	61.7	117	65.4
High	64	20.6	33	18.4

*Lifetime residence*				
Missing data	1		0	
Never rural	178	57.2	95	53.1
Ever rural	133	42.8	84	46.9

**Subjects ≥35 years old**				
*Histology*				
HLNS			72	44.7
HLMC			40	24.8
Other classical HL			35	21.7
Unclassical HL			14	8.7

*Sex*				
Male	1160	53.9	95	59.0
Female	993	46.1	66	41.0

*Age*				
≥35 and <40	134	6.2	43	26.7
≥40 and <45	144	6.7	22	13.7
≥45 and <50	172	8.0	17	10.6
≥50 and <55	243	11.3	22	13.7
≥55 and <60	255	11.8	15	9.3
≥60 and <65	309	14.4	13	8.1
≥65 and <70	342	15.9	12	7.5
≥70 and <75	277	12.9	8	5.0
≥75 and <80	212	9.9	8	5.0
≥80 and <85	50	2.3	1	0.6
≥85 and <100	15	0.7	0	0.0

*Educational level*				
Missing data	0		1	
Low	1067	49.6	65	40.6
Medium	809	37.6	65	40.6
High	277	12.9	30	18.8

*Lifetime residence*				
Missing data	1		1	
Never rural	849	39.5	63	39.4
Ever rural	1303	60.6	97	60.6

HL=Hodgkin's lymphoma.

HLNS=Hodgkin's lymphoma nodular sclerosis.

HLMC=Hodgkin's lymphoma mixed cellularity.

**Table 2:** OR of HL for tobacco smoking and age group

	**Controls *n*=2465**	**All HL, n=340**			**Nodular sclerosis HL, *n*=193**		
	***n***	***n***	**OR^a^**	**(95% CI)**	***n***	**OR^a^**	**(95% CI)**
**Subjects overall**							
*Never smoker*	1095	135			83		
Ever smoker	1361	203	1.33	(1.02–1.74)	108	1.16	(0.82–1.64)
Blond tobacco	572	95	1.20	(0.83–1.73)	61	1.20	(0.76–1.90)
Black tobacco	433	44	1.69	(1.03–2.79)	25	1.76	(0.92–3.38)
Ex-smoker	711	61	1.22	(0.85–1.76)	34	1.31	(0.81–2.11)
Current smoker	650	142	1.39	(1.04–1.87)	74	1.10	(0.75–1.61)

**Subjects <35 years old**							
*Never smoker* *	134	88			63		
Ever smoker	176	91	0.89	(0.60–1.32)	58	0.79	(0.50–1.24)
Blond tobacco	108	52	0.93	(0.57–1.54)	38	0.92	(0.52–1.62)
Black tobacco	24	12	1.15	(0.46–2.87)	9	1.22	(0.43–3.41)
Ex-smoker	32	19	1.05	(0.54–2.05)	16	1.31	(0.63–2.72)
Current smoker	144	72	0.86	(0.57–1.29)	42	0.68	(0.42–1.12)

Cigarettes per day							
<8	55	31	0.98	(0.58–1.68)	22	0.97	(0.52–1.78)
≥8 and <15	51	25	0.77	(0.44–1.36)	19	0.78	(0.41–1.48)
≥15	70	31	0.84	(0.49–1.44)	13	0.51	(0.25–1.05)
*P* trend^b^				0.83			0.83

*Age at start (years)*							
≥18	60	30	0.88	(0.52–1.51)	21	0.93	(0.50–1.73)
>15 and <18	52	32	1.07	(0.62–1.84)	19	0.83	(0.44–1.58)
⩽15	64	27	0.72	(0.41–1.26)	16	0.57	(0.29–1.12)
*P* trend^b^				0.63			0.32

*Duration (years)*							
⩽6	63	28	0.70	(0.41–1.21)	19	0.64	(0.34–1.20)
>6 and ⩽12	62	38	1.01	(0.60–1.68)	27	1.01	(0.56–1.82)
>12	51	23	1.01	(0.53–1.95)	10	0.63	(0.26–1.50)
*P* trend^b^				0.23			0.13

Pack-years							
⩽2	55	29	0.87	(0.50–1.49)	24	0.98	(0.54–1.79)
>2 and <7	59	34	0.97	(0.57–1.62)	21	0.81	(0.44–1.49)
≥7	62	25	0.77	(0.42–1.39)	10	0.43	(0.19–0.96)
*P* trend^b^				0.45			0.08

**Subjects ≥35 years old**							
*Never smoker* **	961	47			20		
Ever smoker	1185	112	1.96	(1.33–2.89)	50	2.17	(1.22–3.88)
Blond tobacco	464	43	1.68	(0.97–2.89)	23	2.09	(0.94–4.68)
Black tobacco	409	32	2.13	(1.13–4.01)	16	2.29	(0.89–5.90)
Ex-smoker	679	42	1.58	(0.99–2.51)	18	1.77	(0.88–3.56)
Current smoker	506	70	2.35	(1.52–3.61)	32	2.56	(1.35–4.85)

*Cigarettes per day*							
<10	308	26	1.81	(1.07–3.06)	11	1.93	(0.87–4.24)
≥10 and <20	390	39	1.95	(1.21–3.14)	16	1.84	(0.89–3.78)
≥20	475	47	2.16	(1.35–3.48)	23	2.90	(1.45–5.82)
*P* trend^b^				0.13			0.49

*Age at start (years)*							
≥20	416	35	1.99	(1.24–3.22)	15	2.23	(1.09–4.55)
>16 and <20	304	30	1.94	(1.16–3.25)	11	1.81	(0.81–4.06)
⩽16	462	47	1.95	(1.21–3.14)	24	2.39	(1.20–4.77)
*P* trend^b^				0.20			0.49

*Duration (years)*							
⩽21	428	35	1.14	(0.70–1.87)	17	1.21	(0.59–2.49)
>21 and ⩽35	383	50	2.91	(1.82–4.67)	21	3.40	(1.66–6.99)
>35	371	27	3.05	(1.74–5.35)	12	4.40	(1.88–10.33)
*P* trend^b^				0.13			0.26

*Pack-years*							
⩽11	391	26	1.14	(0.67–1.93)	12	1.18	(0.53–2.59)
>11 and <30	386	47	2.43	(1.53–3.86)	19	2.33	(1.15–4.73)
≥30	405	39	2.95	(1.77–4.91)	19	4.78	(2.23–10.26)
*P* trend^b^				0.04			0.08

HL=Hodgkin's lymphoma; OR=odds ratio.

* Reference category for all smoking variables among subjects <35 years old.

** Reference category for all smoking variables among subjects ≥35 years old.

a Adjusted for age, sex, educational level, alcohol monthly consumption and centre.

b *P* trend using ever-smokers only (excludes never smokers) and adjusted for the other smoking variables in the table.

**Table 3:** OR of HL for alcohol drinking and age group^a^

	**Controls *n*=1755**	**All HL, *n*=224**			**Nodular sclerosis HL, *n*=137**		
	***n***	***n***	**OR^b^**	**95% CI**	***n***	**OR^b^**	**95% CI**
**Subjects overall**							
*Never regular drinker*	876	141			88		
Ever regular drinker	866	81	0.61	(0.43–0.87)	47	0.58	(0.37–0.92)
Beer drinker	507	57	0.63	(0.41–0.94)	34	0.60	(0.35–1.02)
Wine drinker	645	43	0.57	(0.37–0.88)	23	0.60	(0.34–1.06)
Spirits drinker	284	42	0.78	(0.50–1.22)	25	0.68	(0.38–1.20)

**Subjects <35 years old**							
Never regular drinker*	119	83			61		
Ever regular drinker	96	40	0.63	(0.37–1.09)	28	0.62	(0.33–1.16)
Beer drinker	69	33	0.76	(0.41–1.39)	23	0.78	(0.38–1.59)
Wine drinker	41	12	0.49	(0.22–1.10)	7	0.44	(0.16–1.19)
Spirits drinker	46	24	0.78	(0.41–1.51)	17	0.70	(0.33–1.50)

*Monthly consumption (g)*							
⩽66	6	3	0.79	(0.18–3.51)	2	1.13	(0.18–7.17)
>66 and ⩽341	13	4	0.56	(0.16–2.01)	2	0.46	(0.09–2.50)
>341	24	9	0.72	(0.27–1.89)	7	0.91	(0.29–2.80)
*P* trend^c^				0.17			0.44

*Age at start (years)*							
≥18	38	19	0.75	(0.38–1.47)	11	0.57	(0.25–1.30)
≥16 and <18	35	13	0.50	(0.23–1.12)	10	0.54	(0.22–1.33)
<16	22	6	0.46	(0.17–1.26)	6	0.76	(0.26–2.22)
*P* trend^c^				0.52			0.64

*Duration (years)*							
⩽7	35	15	0.57	(0.27–1.19)	13	0.62	(0.28–1.39)
>7 and ⩽13	35	12	0.47	(0.21–1.05)	6	0.28	(0.10–0.81)
>13	25	11	1.01	(0.40–2.56)	8	1.57	(0.52–4.75)
*P* trend^c^				0.03			0.18

*Lifetime consumption (kg)*							
<16	27	8	0.44	(0.18–1.06)	4	0.29	(0.09–0.92)
≥16 and <61	31	14	0.66	(0.31–1.41)	10	0.69	(0.29–1.65)
≥61	36	16	0.76	(0.35–1.64)	13	0.96	(0.40–2.33)
*P* trend^c^				0.07			0.16

**Subjects ≥35 years old**							
Never regular drinker**	757	58			27		
Ever regular drinker	770	41	0.58	(0.35–0.94)	19	0.52	(0.26–1.04)
Beer drinker	438	24	0.51	(0.28–0.92)	11	0.42	(0.18–0.98)
Wine drinker	604	31	0.58	(0.34–1.01)	16	0.67	(0.32–1.42)
Spirits drinker	238	18	0.71	(0.38–1.36)	8	0.57	(0.23–1.43)

*Monthly consumption (g)*							
⩽215	121	5	0.52	(0.20–1.38)	3	0.58	(0.16–2.08)
>215 and ⩽734	249	13	0.63	(0.32–1.24)	7	0.64	(0.25–1.60)
>734	274	19	0.66	(0.35–1.26)	7	0.47	(0.18–1.25)
*P* trend^c^				0.09			0.40

*Age at start (years)*							
>22	216	12	0.72	(0.36–1.42)	7	0.82	(0.33–2.05)
>18 and ⩽22	203	7	0.44	(0.19–1.04)	3	0.41	(0.12–1.48)
⩽18	320	22	0.63	(0.34–1.15)	9	0.45	(0.18–1.10)
*P* trend^c^				0.88			0.31

*Duration (years)*							
⩽29	238	20	0.53	(0.29–0.97)	10	0.47	(0.20–1.11)
>29 and ⩽44	266	15	0.73	(0.37–1.44)	5	0.53	(0.18–1.57)
>44	253	6	0.51	(0.19–1.38)	4	0.76	(0.21–2.77)
*P* trend^c^				0.60			0.40

*Lifetime consumption (kg)*							
<143	221	16	0.72	(0.39–1.33)	9	0.76	(0.33–1.76)
≥143 and <425	259	14	0.55	(0.28–1.09)	5	0.38	(0.13–1.09)
≥425	259	11	0.50	(0.23–1.07)	5	0.44	(0.15–1.33)
*P* trend^c^				0.07			0.23

CI=confidence interval; HL=Hodgkin's lymphoma; OR=odds ratio.

* Reference category for all alcohol variables among subjects <35 years old.

** Reference category for all alcohol variables among subjects ≥35 years old.

a Excluding data from Germany.

b Adjusted for age, sex, educational level, smoking status and centre.

c *P* trend using ever-regular drinkers only (excludes never-regular drinkers) and adjusted for the other alcohol variables in the table.
